# Latest trends in *Leishmania infantum* infection in dogs in Spain, Part I: mapped seroprevalence and sand fly distributions

**DOI:** 10.1186/s13071-020-04081-7

**Published:** 2020-04-21

**Authors:** Rosa Gálvez, Ana Montoya, Israel Cruz, Carlos Fernández, Oihane Martín, Rocío Checa, Carmen Chicharro, Silvia Migueláñez, Valentina Marino, Guadalupe Miró

**Affiliations:** 1 Grupo de Investigación Epicontrol-Carnívoros, Departamento de Sanidad Animal, Facultad de Veterinaria, Universidad Complutense de Madrid, Madrid, Spain; 2 National School of Public Health, Instituto de Salud Carlos III, Madrid, Spain; 3 Microbiology Department, Hospital Universitario Ramón y Cajal, Madrid, Spain; 4 Parasitology Unit, Centro Nacional de Microbiología, Instituto de Salud Carlos III, Majadahonda, Spain

**Keywords:** *Leishmania infantum*, Canine leishmaniosis, Seroprevalence, Risk factors, Spain, Immunofluorescence antibody test, Phlebotomine sand fly, Bioclimatic belt

## Abstract

**Background:**

This report describes *L. infantum* infection seroprevalence in dogs in Spain through data obtained from peer-reviewed literature and a cross-sectional serological survey assessing epidemiological and habitat variables as risk factors for infection. The study also provides preliminary sand fly species distribution data and indicates factors affecting their distribution and density.

**Methods:**

Three different studies were conducted in Spain: (i) a peer-reviewed literature seroprevalence survey (1985–2019); (ii) a cross-sectional serological survey (2011–2016); and (iii) a preliminary entomological survey (2013–2014). In the cross-sectional serological survey, 1739 dogs from 74 different locations including 25 Spanish provinces were tested for *L. infantum* by indirect immunofluorescence antibody test (IFAT) (antibody titre ≥ 1:100). Seroprevalence of *L. infantum* infection was analysed by province and bioclimatic zone. Statistics were used to analyse relationships between several dog- and environment-related variables and *L. infantum* seroprevalence. In parallel, during 2013–2014, sand flies were collected across the Iberian Peninsula and the Balearic Islands using CDC light traps to examine relationships between habitat-related factors and sand fly species densities (number of sand flies per trap per hour).

**Results:**

The literature review revealed that the provinces showing the highest seroprevalence were Balearic Islands (57.1%), Ourense (35.6%), Málaga (34.6%) and Cáceres (34.2%), and those showing the lowest seroprevalence were Vizcaya (0%), Cantabria (2.0%) and Álava (3.3%). In our survey, anti-*Leishmania* IgG antibodies were detected in 176 of the 1739 dogs rendering a seroprevalence of 10.12%. Percentage seroprevalence distributions significantly varied among bioclimatic belts. Seropositivity for *L. infantum* was related to size (large breed dogs *versus* small) and were significantly higher in younger dogs (≤ 1 years-old). In the entomological survey, 676 sand flies of five species were captured: 562 (83.13%) *Phlebotomus perniciosus*; 64 (9.47%) *Sergentomyia minuta*; 38 (5.62%) *P. ariasi*: 6 (0.89%) *P. sergenti*; and 6 (0.89%) *P. papatasi*. *Phlebotomus perniciosus* showed a greater density in the thermo-Mediterranean than in the meso-Mediterranean zone. Densities of *S. minuta* and *P. ariasi* were significantly higher in rural habitats.

**Conclusions:**

This updated seroprevalence map of *L. infantum* infection in dogs in Spain defines non-endemic, hypoendemic, endemic and hyperendemic areas, and confirms *P. perniciosus* as the most abundant sand fly vector in Spain.
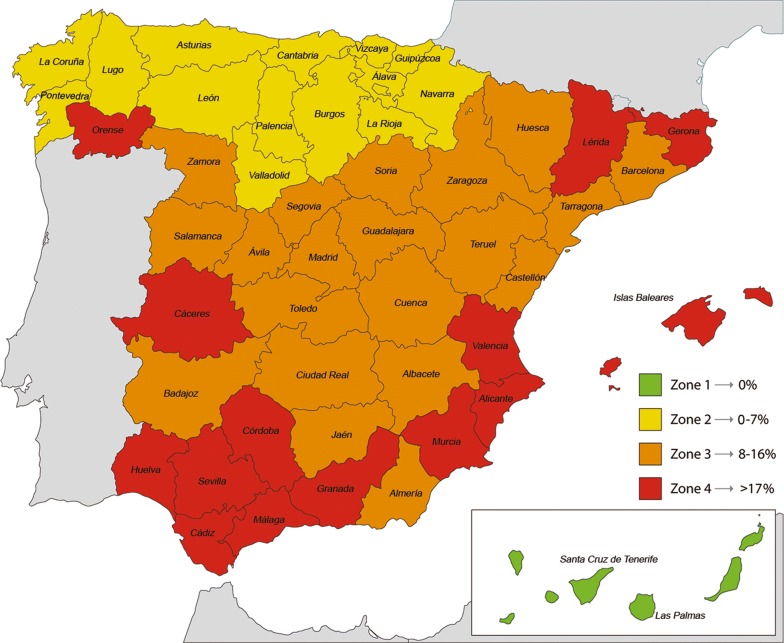

## Background

Leishmaniosis caused by *Leishmania infantum* is a widespread zoonotic disease that may be transmitted to animals and humans by their vectors, blood-sucking phlebotomine sand flies [1, 2]. Other non-sand fly routes of *L. infantum* transmission include vertical and horizontal routes (from blood donors, venereal transmission and direct dog-to-dog transmission through bites or wounds) [3]. In endemic areas, a population subset with subclinical infection acts as a disease reservoir [3]. In Spain, CanL is an endemic and dynamic disease with an overall seroprevalence and transmission risk that vary according to local environmental and climatic conditions [4, 5]. In the Mediterranean basin, the dog is the main reservoir for *L. infantum*, and it is estimated that close to 2.5 million dogs are infected [6]. In Spain, CanL shows a broad seroprevalence range varying according to the geographical area [7]. In the northern provinces of the Iberian Peninsula traditionally classified as non-endemic, seroprevalence is lower [8]. Climate and environmental changes provoked by human activities may have caused the expansion of *L. infantum* infection in dogs towards such areas historically considered disease-free [9]. Regarding sand fly status in Spain, *Sergentomyia minuta* is the most abundant species, followed by two vector species of *L. infantum*: *P. perniciosus*, which is more widespread and less affected by climatic conditions, and *P. ariasi*, which shows a preference for humid, cold areas [2].

There is great variability in how *L. infantum* manifests in a dog due to both individual factors (e.g. breed, age, immune status) and environmental factors (e.g. climate, land use) [3, 10, 11].

Control strategies should be based on local epidemiological information [1]. The updated data provided here on the seroprevalence of CanL and on the ecology of sand fly vectors in Spain will be useful for the design of targeted control measures.

This study is Part I of a larger investigation addressing the situation of CanL in Spain. In Part II, we examined how CanL is currently managed *via* a multicentre questionnaire completed by veterinarians and compared the data obtained with a similar survey conducted in 2005.

## Methods

### Study area

The study area was mainland Spain and the Balearic and Canary Islands. Nine bioclimatic zones have been traditionally defined for the Iberian Peninsula and Balearic Islands [12]. Five of these areas occupying 46 × 10^6^ ha were surveyed: supratemperate and mesotemperate within the Eurosiberian region, and supra-Mediterranean, meso-Mediterranean and thermo-Mediterranean within the Mediterranean region. The zones not surveyed were the four highest regions occupying 1 × 10^6^ ha (alpine and subalpine in the Eurosiberian region, and cryoro-Mediterranean and oro-Mediterranean in the Mediterranean region; mean altitudes of 2396, 1882, 2548 and 1757 meters above sea level, respectively) because climatic conditions are not suitable for sand fly development.

### *Leishmania infantum* seroprevalence study

#### Literature review

Scientific works published from 1985 to 2019 reporting CanL seroprevalences for mainland Spain and the Balearic Islands were identified. Inclusion criteria were seroprevalence studies conducted on randomly sampled dog populations in which the humoral response was assessed by detecting antibodies. According to the antibody titre cut-off established in each study, seroprevalence ranges were calculated for each province. These data were used to prepare a seroprevalence map of *L. infantum* in the dog.

#### Cross-sectional serological survey

During 2011–2016, a cross-sectional seroprevalence study was performed in 25 Spanish provinces without taking into account the clinical status of dogs. Epidemiological variables recorded included geographical location, habitat, age, sex, breed, weight, travel history and presence of clinical signs. A 5 ml blood sample was obtained from each dog by cephalic venipuncture and sera were separated and kept at − 20 °C until analysis. Serodiagnosis was conducted by detecting specific antibodies against *L. infantum* using the indirect immunofluorescence antibody test (IFAT) for anti-*Leishmania*-specific immunoglobulin G (IgG) antibodies according to standard procedures [13]. Serological analyses were conducted at the Pet Parasite Lab (Animal Health Department Veterinary Faculty, UCM, Spain). The cut-off indicating a positive result was 1:100. Seroprevalence was calculated as the percentage of dogs testing positive for anti-*L. infantum* antibodies.

### Entomological survey

Sand flies were collected from the wild using CDC light traps set up in the afternoon and recovered early in the morning. Sand flies were trapped in 2013–2014 seasons (from May to October). Captured sand flies were transferred to labelled 1.5 ml tubes containing 70% ethanol and identified to the species level. Females were cleared in Mark-André medium [14] and mounted on glass slides in Hoyerʼs medium [15]. Sand flies were identified according to taxonomic keys [16]. Sand fly densities were calculated according to the following formula: Sand fly density = No. of sand flies/(No. of traps × hours).

Sites were geocoded by locality using ArcGis Pro v.2.3.3 [Environmental Systems Research Institute (ESRI), Redlands, CA, USA].

### Statistical analysis

For the cross-sectional serological survey, Chi-square test and Student’s t-test were used to identify significant associations between *L. infantum* seroprevalence and age, sex, breed, size, habitat, use given and bioclimate. Seroprevalence was calculated separately for every province and bioclimatic zone. Kruskal-Wallis and Wilcoxon signed rank tests were used to examine relationships between sand fly species density (number of sand flies per trap per hour) and the variables bioclimatic zone, habitat and presence of animals (domestic, farm or wild fauna). Significance was set at P ≤ 0.05. All statistical tests were performed using the SPSS 25 package (SPSS Inc., Chicago, IL, USA).

## Results

**Fig. 1:**
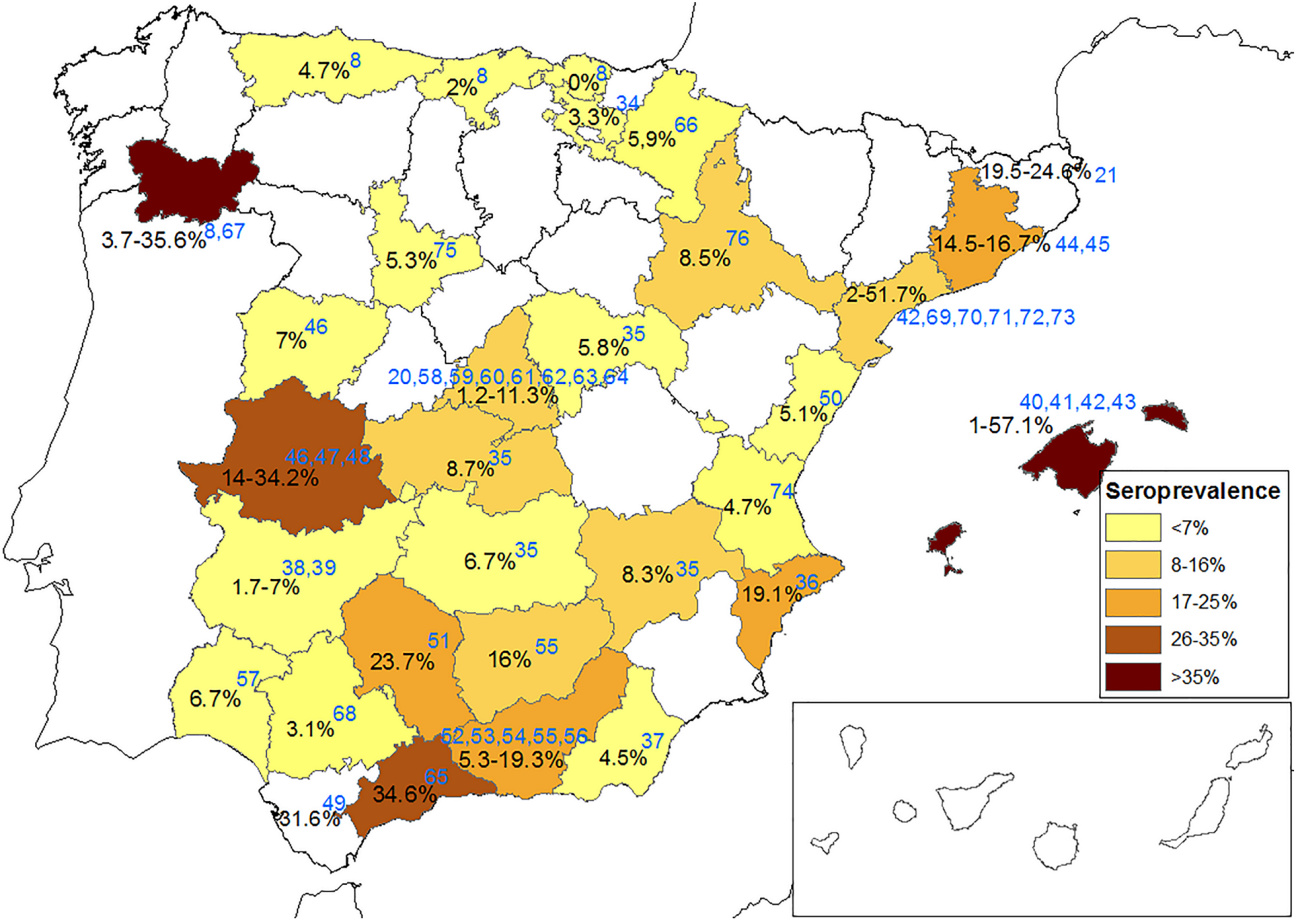
Seroprevalence of canine *L. infantum* infection in Spain by province based on a review of the literature published from 1985 to 2019. *References*: Miró et al. [8]; Gálvez et al. [20]; Vélez et al. [21]; Sáez de Santamaría et al. [34]; Benito et al. [35]; Alonso et al. [36]; Sanchís Marín et al. [37]; Rosado et al. [38]; Asencio et al. [39]; Seguí [40]; Pujol et al. [41]; Solano-Gallego et al. [42]; Alcover et al. [43]; Botet et al. [44]; Corachan et al. [45]; Encinas Grandes et al. [46]; Nieto et al. [47]; Rosado et al. [48]; Morales-Yuste et al. [49]; Arnedo Pena et al. [50]; Martínez-Cruz et al. [51]; Reyes Magaña et al. [52, 53]; Acedo Sánchez et al. [54, 55]; Martín-Sánchez et al. [56]; Lepe et al. [57]; Celaya [58]; Amela et al. [59]; Castañeda et al. [60, 61]; García Nieto et al. [62]; Miró et al. [63, 64]; Morillas et al. [65]; Sesma et al. [66]; Amusátegui et al. [67]; Ariza-Astolfi et al. [68]; Portús et al. [69]; Fisa et al. [70, 71]; Fisa et al. [72, 73]; Benito-Hernández et al. [74]; Couto et al. [75]; Castillo Hernández et al. [76]

Forty-one scientific works reporting CanL seroprevalences for mainland Spain and the Balearic Islands were identified. The techniques used were IFI, ELISA and rapid tests. The review of reported seroprevalences of *L. infantum* revealed that of the 50 Spanish provinces there are published data for 31 of them. The provinces showing the highest seroprevalence were Balearic Islands (57.1%), Ourense (35.6%), Málaga (34.6%) and Cáceres (34.2%), followed by Gerona (24.6%), Córdoba (23.7%), Granada (19.3%) and Alicante (19.1%). Provinces showing the lowest seroprevalence were Vizcaya (0%), Cantabria (2%) and Álava (3.3%). Seroprevalence reported for the remaining provinces varied between 5–16% (Table 1 and Fig. 1).

**Table 1:** Seroprevalence of canine *L. infantum* infection in Spain by province based on a review of literature published from 1985 to 2019

Province	Seroprevalence (%)				Seroprevalence range (%)	*n*	Reference
	IFAT	ELISA	IFAT & ELISA	Commercial kit			
Álava	3.3	–	–	–	3.3	na	Sáez de Santamaría et al. [34]
Albacete	–	–	8.3	–	8.3	232	Benito et al. [35]
Alicante	–	19.1	–	–	19.1	807	Alonso et al. [36]
Almería	4.5	–	–	–	4.5	286	Sanchís Marín et al. [37]
Asturias	4.7	–	–	–	4.7	171	Miro et al. [8]
Badajoz	–	7.0	–	–	1.7–7.0	na	Rosado et al. [38]
	–	–	–	1.7		na	Asencio et al. [39]
Balearic Is.	–	1.0	–	–	1.0–57.1	813	Seguí [40]
	35.2	38.4	–	–		353	Pujol et al. [41]
			29.3			300	Solano-Gallego et al. [42]
	57.1	35.7	–	52.4		121	Alcover et al. [43]
Barcelona	14.5	–	–	–	14.5–16.7	617	Botet et al. [44]
	16.7	–	–	–		466	Corachan et al. [45]
Cáceres	15.0	–	–	–	14.0–34.2	433	Encinas Grandes et al. [46]
	14.0	–	–	–		381	Nieto et al. [47]
	–	34.2	–	–		240	Rosado et al. [48]
Cádiz	31.6				31.6	98	Morales-Yuste et al. [49]
Cantabria	2.0	–	–	–	2.0	100	Miró et al. [8]
Castellón	5.1	–	–	–	5.1	118	Arnedo Pena et al. [50]
Ciudad Real	6.7	–	–	–	6.7	232	Benito et al. [35]
Córdoba	–	–	23.7	–	23.7	540	Martínez-Cruz et al. [51]
Granada	8.8	–	–	–	5.3–19.3	1503	Reyes Magaña et al. [52]
	19.3	–	–	–		na	Reyes Magaña et al. [53]
	5.3	–	–	–		615	Acedo-Sánchez et al. [54]
	16.1	–	–	–		1374	Acedo-Sánchez et al. [55]
	13.0	–	–	–		439	Martín-Sánchez et al. [56]
Girona	–	19.5–24.6	–	–	19.5–24.6	168	Vélez et al. [21]
Guadalajara	–	–	5.8	–	5.8	232	Benito et al. [35]
Huelva	6.7	–	–	–	6.7	702	Lepe et al. [57]
Jaén	16.0	–	–	–	16.1	1374	Acedo-Sánchez et al. [55]
Madrid	4.7	–	–	–	1.2–11.3	473	Celaya [58]
	5.2	–	–	–		591	Amela et al. [59]
	4.7	–	–	–		235	Castañeda et al. [60]
	11.3	–	–	–		278	Castañeda et al. [61]
	8.6	–	–	–		775	García Nieto et al. [62]
	7.8	–	–	–		1803	Miró et al. [63]
	8.1	–	–	–		1076	Gálvez et al. [20]
	1.2	–	–	–		1372	Miró et al. [64]
Málaga	34.6	–	–	–	34.6	344	Morillas et al. [65]
Navarra	5.9	–	–	–	5.9	653	Sesma et al. [66]
Ourense	3.7	–	–	–	3.7–35.6	479	Amusátegui et al. [67]
	35.6	–	–	–		101	Miró et al. [8]
Salamanca	7.0	–	–	–	7.0	433	Encinas Grandes et al. [46]
Sevilla	3.1	–	–	–	3.1	1000	Ariza-Astolfi et al. [68]
Tarragona	2.0	–	–	–	2.0–51.7	895	Portús et al. [69]
	15.0	–	–	–		104	Fisa et al. [70]
	10.2	–	–	–		902	Fisa et al. [71]
	–	–	10.2	–		107	Fisa et al. [72]
	–	–	5.7	–		2110	Fisa et al. [73]
			51.7			116	Solano-Gallego et al. [42]
Toledo	8.7	–	–	–	8.7	232	Benito et al. [35]
Valencia	–	–	4.7	–	4.7	215	Benito-Hernández et al. [74]
Valladolid	–	–	–	5.3	5.3	131	Couto et al. [75]
Vicaya	0	–	–	–	0	47	Miro et al. [8]
Zaragoza	8.5	–	–	–	8.5	1572	Castillo Hernández et al. [76]

*Abbreviation*: n, number of dogs surveyed; na, not applicable

**Fig. 2:**
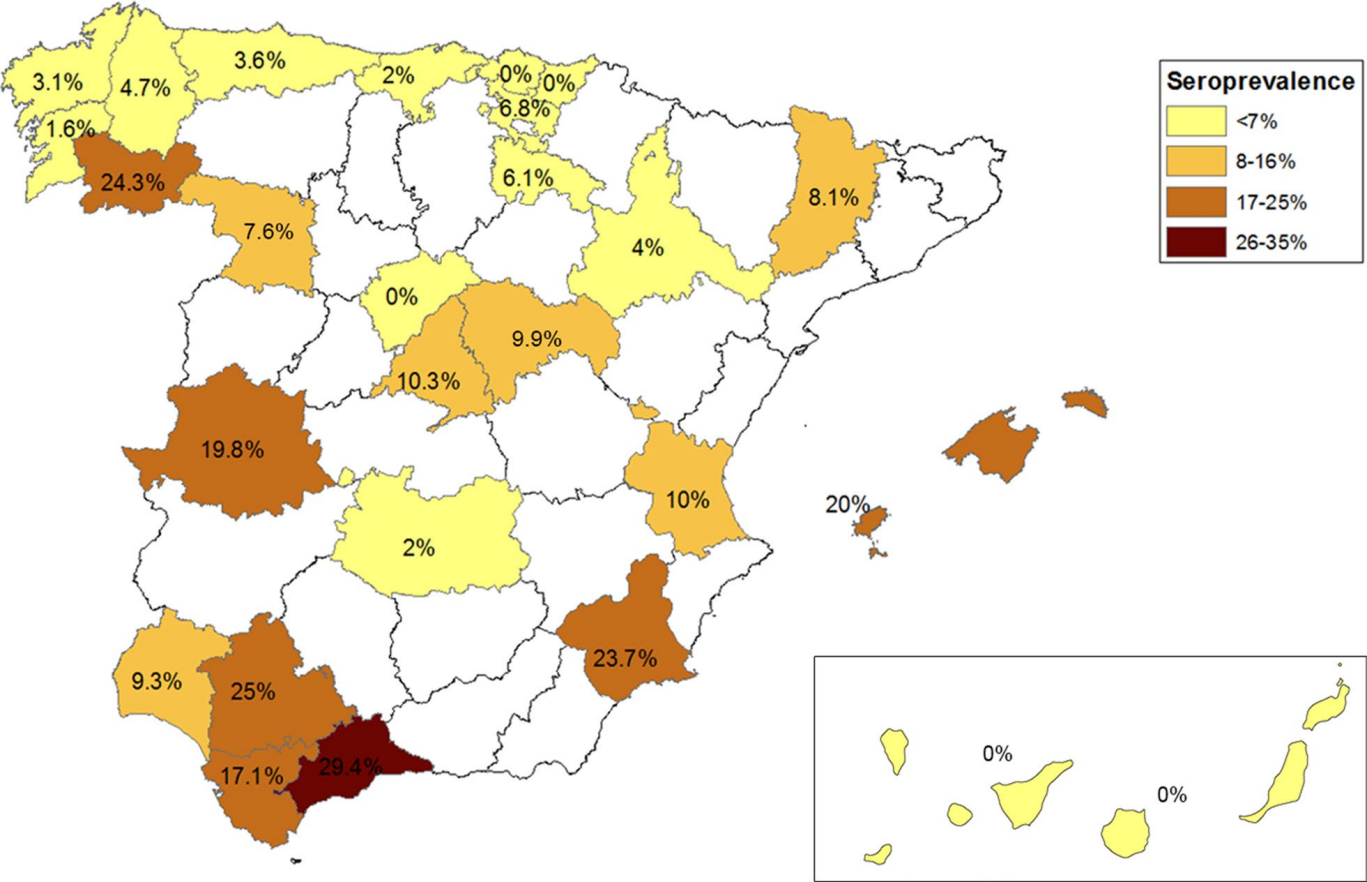
Seroprevalence of canine *L. infantum* infection in Spain by province based on the cross-sectional serological survey conducted during 2011–2016

The features of the cross-sectional seroprevalence study dog population are provided in Table 2. The animals surveyed (*n* = 1739) were included into the following dog populations: (i) municipal animal shelter (35%, 608 stray dogs); (ii) hunting animal shelter (47%, 813 hunting dogs, 2 pets, 1 guard dog and 1 shepherd dog); (iii) housed dogs (13.3%, 229 pets and 2 guard dogs); and (iv) farms (4.7%, 21 shepherd dog, 11 pets, 8 hunting dogs and 1 guard dog). The study was conducted in 74 different locations in 25 Spanish provinces (see Table 3 and Fig. 2). Eleven new provinces without literature data were surveyed: A Coruña, Guipúzcoa, La Rioja, Las Palmas, Lérida, Lugo, Murcia, Pontevedra, Santa Cruz de Tenerife, Segovia, Valencia and Zamora.

**Table 2:** Features of the dog population in the cross-sectional survey of canine leishmaniosis seroprevalence (2011–2016)

Variables	*n* (%)	No. positive (%)
Sex		
Female	732 (42)	65 (9)
Male	991 (58)	111 (11)
Age group		
< 1 year	173 (11)	31 (18)
1–2 years	473 (29)	32 (7)
3–4 years	372 (23)	36 (10)
5–6 years	284 (17)	35 (12)
7–8 years	181 (11)	18 (10)
> 9 years	154 (9)	10 (6)
Breed size		
Large (> 20 kg)	989 (60)	121 (12)
Medium (10–19 kg)	457 (28)	34 (7)
Small (< 10 kg)	203 (12)	12 (6)
Dog populations		
Municipal animal shelter	608 (35)	63 (10)
Hunting animal shelter	817 (47)	79 (10)
In the home	231 (13)	29 (13)
Farm	83 (5)	5 (6)
Habitat		
Peri-urban	506 (29)	506 (29)
Rural	1048 (60)	1048 (60)
Urban	185 (11)	185 (11)
Bioclimate		
Supratemperate	93 (5)	93 (5)
Mesotemperate	519 (30)	519 (30)
Supra-mediterranean	526 (30)	526 (30)
Meso-mediterranean	462 (27)	462 (27)
Thermo-mediterranean	139 (8)	139 (8)
Use given		
Stray dog	608 (35)	63 (10)
Hunting dog	821 (47)	80 (10)
Pet	242 (14)	28 (12)
Goat dog	64 (4)	4 (6)
Guard dog	4 (4)	1 (25)

*Abbreviation*: n, number of dogs

**Table 3:** Seroprevalence of canine *L. infantum* infection in Spain by province based on the cross-sectional serological survey conducted during 2011–2016

Province	Seroprevalence			Localities	Bioclimatic zone
	*n*	%	No. positive		
Álava	161	6.8	11	Arlucea, Artziniega, Madaria, Marsoño, Menoio, Noitegui, Quintana, Orbiso, Ollabarre, Respaldiza, San Román de Campezo, Sojo, Soxoguti	Mesotemperate
Asturias	110	3.6	4	Langreo, Serín	Mesotemperate
Baleares (Ibiza)	40	20.0	8	Sant Andoni de Portmany, Sant Joan de Labritja	Thermo-Mediterranean
Cáceres	91	19.8	18	Cañamero, Madrigal de la Vera	Meso-Mediterranean
Cádiz	35	17.1	6	Los Barrios	Thermo-Mediterranean
Cantabria	100	2.0	2	Torrelavega	Mesotemperate
Ciudad Real	51	2	1	Puertollano	Meso-Mediterranean
Coruña	32	3.1	1	La Coruña, Laracha	Mesotemperate, Supratemperate
Guadalajara	81	9.9	8	El Casar, Espinosa de Henares, Torremocha de Jadraque	Supra-Mediterranean, meso-Mediterranean
Guipúzcoa	14	0	0	Antoñana, Izoria, Menagaray, Santurce	Mesotemperate
Huelva	97	9.3	9	Valverde del Camino	Meso-Mediterranean
La Rioja	66	6.1	4	Arnedo, Herce, Prejano	Meso-Mediterranean
Las Palmas	104	0	0	Arguineguín	Inframacaronesian
Lérida	37	8.1	3	Alcarras	Supra-Mediterranean
Lugo	85	4.7	4	Lugo, Guitiriz	Supratemperate
Madrid	224	10.3	23	Fuenlabrada, Móstoles, Navalcarnero, Peralejo, Serrada de la Fuente, Villalba, Villavicioesa, Las Zorreras	Supra-Mediterranean
Málaga	17	29.4	5	Alhaurín el Grande, Fuengirola, Mijas,	Thermo-Mediterranean
Murcia	59	23.7	14	Achivel, Molina de Segura, Moratalla	Meso-Mediterranean, thermo-Mediterranean
Orense	152	24.3	37	Allariz, Orense	Supra-Mediterranean
Pontevedra	64	1.6	1	Vigo	Mesotemperate
Santa Cruz de Tenerife	31	0	0	Tierra blanca	Inframacaronesian
Segovia	14	0	0	Fuentepelayo y Cabanillas, Revenga	Supra-Mediterranean
Sevilla	32	25.0	8	Castilblanco de los Arroyos	Thermo-Mediterranean
Valencia	40	10.0	4	Utiel	Meso-Mediterranean
Vizcaya	46	0	0	Santa Cruz, Hueto Abajo/Arriba	Mesotemperate
Zamora	66	7.6	5	Arquelinos, Bretó, Pajares, Piedrahita de Castro, Revenillos del Campo, Santovenia, Villarrín de Campos	Supra-Mediterranean
Zaragoza	25	4	1	Codos	Supra-Mediterranean

*Abbreviation*: n, number of dogs

Overall, 176 of the 1739 dogs examined tested positive for *L. infantum* (10.1%). Significant differences were detected between longer deviated seroprevalence values in relation to bioclimatic belt (*χ*^2^ = 51.9968, *df* = 4, *P* < 0.0001): supratemperate (5.4%), mesotemperate (3.5%), supra-Mediterranean (13.1%), meso-Mediterranean (11.9%), and thermo-Mediterranean (20.9%).

Significant differences were also detected when comparing seroprevalences by age group (*χ*^2^ = 21.5852, *df* = 5, *P* = 0.0006) and seroprevalence rates were higher in the younger dogs (< 1 year-old). Dog size data also revealed significant differences (*χ*^2^ = 12.4160, *df* = 2, *P* = 0.0020) and seroprevalence was higher in larger-sized dogs. Significant differences were observed when comparing seroprevalences for the different habitats (*χ*^2^ = 10.5837, *df* = 2, *P *= 0.005) and seroprevalence was higher in rural habitats. No differences were observed according to sex (*χ*^2^ = 2.4730, *df* = 1, *P* = 0.1158) or use category given to the dogs (*χ*^2^ = 2.7534, *df* = 4, *P* = 0.5999).

**Fig. 3:**
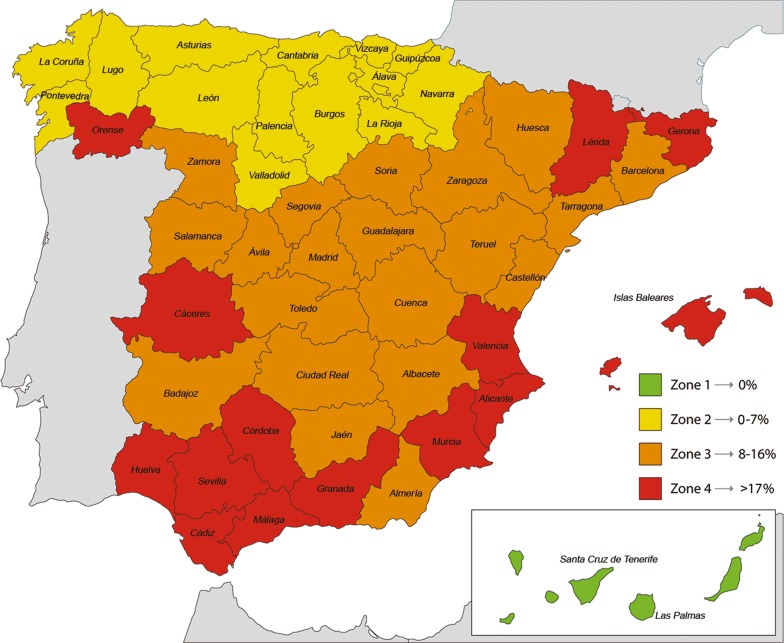
Seroprevalence of canine *L. infantum* infection in Spain: compendium of Figs. 1 and 2. Zone 1 (non-endemic, low risk), Zone 2 (hypoendemic, intermediate risk), Zone 3 (endemic, intermediate-high risk) and Zone 4 (hyperendemic, high risk)

Cross-sectional survey data, together with published data for seroprevalence of *L. infantum* reveal that there are available data for 42 of the 50 provinces in Spain. A map of overall *L. infantum* infection seroprevalence was constructed (Fig. 3) by combining the data obtained in the literature review (Fig. 1) and the present survey (Fig. 2) based on seroprevalence records and the new areas reported here. This map provides seroprevalence data extrapolated to the entire country defining zones classified according to the seroprevalence range as: Zone 1 (non-endemic, low risk); Zone 2 (hypoendemic, intermediate risk); Zone 3 (endemic, intermediate-high risk); and Zone 4 (hyperendemic, high risk).

**Fig. 4:**
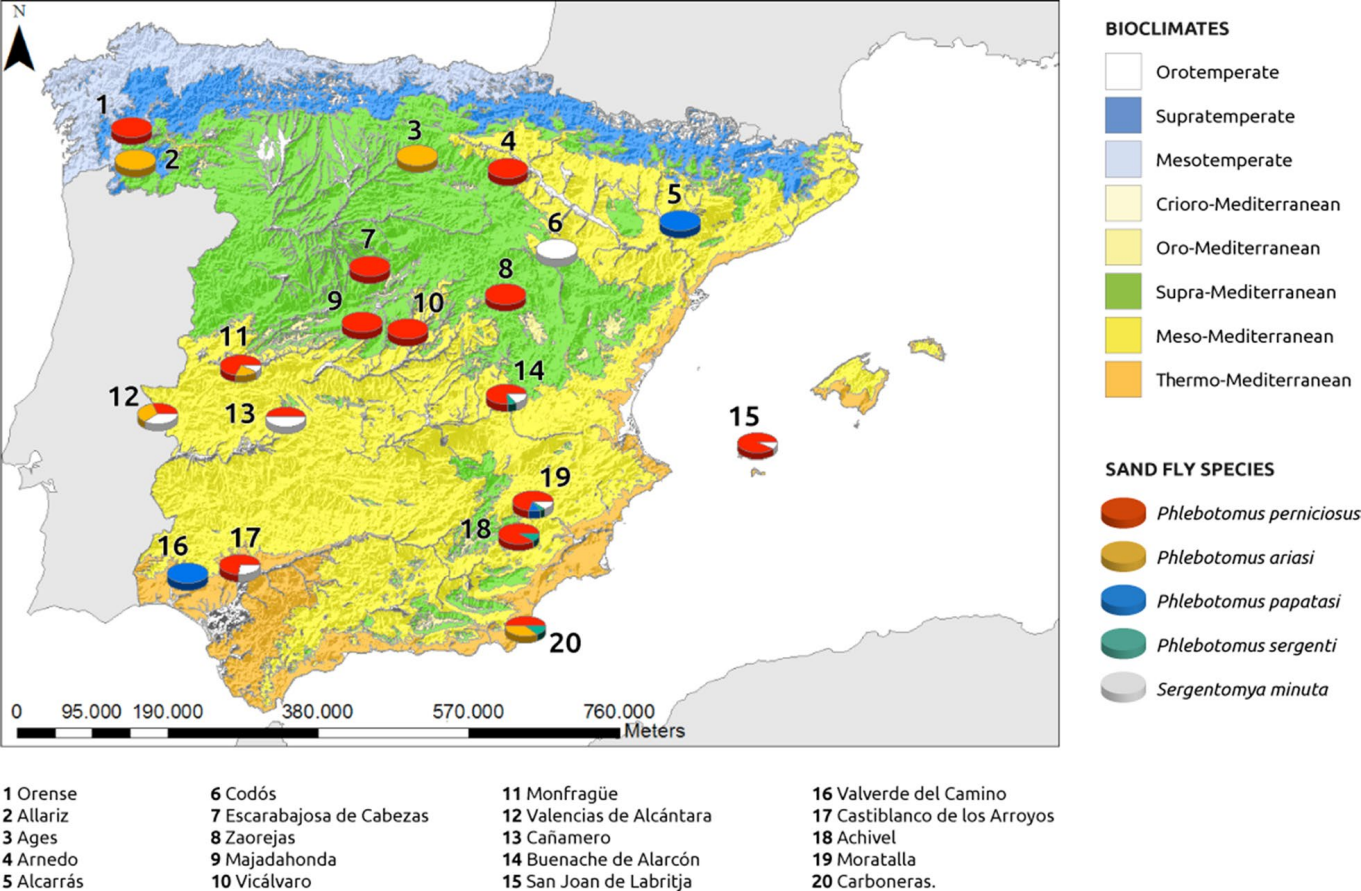
Sites surveyed for sand flies across the Iberian Peninsula and Balearic Islands shown on a bioclimatic zone map

In the entomological survey conducted, fifty sites were sampled in 20 localities across the Mediterranean region (see Additional file 1: Table S1 and Fig. 4). The number of sites surveyed in each bioclimatic area was proportional to the size of the zone such that the largest bioclimatic belts, meso-Mediterranean and supra-Mediterranean were the most surveyed (22 sampling sites each) followed by the thermo-Mediterranean belt (6 sampling sites). A total of 676 specimens of five species were captured and further identified as follows: 562 (83.13%) *P. perniciosus*; 64 (9.47%) *S. minuta*; 38 (5.62%) *P. ariasi*; 6 (0.89%) *P. sergenti*; and 6 (0.89%) *P. papatasi*. Sand fly sites were georeferenced and depicted as a pie chart reflecting the proportion of each sand fly species (Fig. 4). Traps were set in rural (20 sites, 60%) and periurban habitats (20 sites, 40%).

Over the 2-year study period, densities of *S. minuta* (*Z* = − 2.7485, *P* = 0.0084) and *P. ariasi* (*Z* = − 2.2811, *P* = 0.0269) were increased in rural habitats. *Phlebotomus perniciosus* showed a greater density increase in the thermo-Mediterranean compared with the meso-Mediterranean zone (*Z* = − 2.75663, *P* = 0.00584). In addition, the densities of the vector species (*P. perniciosus* and *P. ariasi*) (*Z* = − 2.58737, P = 0.00967) and of all phlebotomines (*Z* = − 2.70698, *P* = 0.00679) also rose significantly in the thermo-Mediterranean compared with the meso-Mediterranean belt. New records were also observed for *P. ariasi* in Burgos, *P. papatasi* in Huelva and *P. perniciosus* in Segovia.

## Discussion

In a CanL endemic country like Spain with high densities of sand fly vectors and reservoirs, *L. infantum* infection spreads quickly amongst dog populations. The overall seroprevalence of *L. infantum* infection in the dogs surveyed was 10.1% (176/1739 animals), taking ≥ 1:100 as the cut-off antibody titre. In areas where CanL is endemic, such a seroprevalence value of around 10% represents both dogs that develop the disease and a fraction of clinically healthy but persistently infected dogs [3]. In fact, CanL is only the tip of the iceberg in endemic areas, where part of the population is exposed and becomes infected without showing clinical evidence of disease [11]. In the survey, twelve provinces not sampled before were included. In future studies, canine *L. infantum* seroprevalence distributions need to be determined for the as yet unsampled provinces (in alphabetical order): Ávila, Burgos, Cuenca, Huesca, León, Palencia, Soria and Teruel. Differences between seroprevalence distributions in relation to bioclimatic belt were significant. The thermo-Mediterranean belt yielded the highest seroprevalence (20.9%) and may be considered of high-risk; the belts supra-Mediterranean (13.12%) and meso-Mediterranean (11.9%) of intermediate risk; and the belts supratemperate (5.4%) and mesotemperate (3.5%) of low risk.

In northern Spain, where the seroprevalence was low compared to the rest of the Iberian Peninsula, we found elevated prevalence (24.3%) in Ourense [8]. Despite the lower seroprevalence in northern Spain, the climatic conditions of the Galician province of Ourense should be highlighted as highly suitable for the presence of *L. infantum* [8]. This province features exceptionally adequate climate conditions for the expansion of leishmaniosis and it belongs to the supra-Mediterranean and not the temperate bioclimatic zone, like the northern region of Spain. Indeed, data from northern areas of Spain have already shown the expansion of *L. infantum* infection [8, 17].

According to Figs. 1 and 2, reported *L. infantum* seroprevalence in Spain ranges from 2% to 57.1% depending on the geographical region. *Leishmania infantum* seroprevalence variation between Spanish provinces might be explained by geographical differences in climate and seasonality associated with the bioclimatic and ecological requirements of the sand fly vectors [18]. In the European Union there is a zoonotic cutaneous and visceral leishmaniosis caused by *L. infantum* throughout the Mediterranean region [9]. It has been proposed that environmental changes and global warming are having an impact on the geographical distribution of CanL infection and its vectors all over Europe [9, 18, 19]. The map in Fig. 3 shows a snapshot of *L. infantum* seroprevalence that shows the northward emergence of CanL in Spain. These compendium maps of *L. infantum* seroprevalence will be useful for the implementation of control programmes. While it is desirable to standardize the data source of the surveys upon which we created Fig. 3, we have to take into account that they can present many varying factors such as dog selection procedures, serological techniques and antibody titre cut-offs used, different periods (from 1985 up to date) and sample sizes, among many others.

In the present cross-sectional study, differences were detected in *L. infantum* infection seroprevalence according to animal age such that seroprevalences were significantly higher among younger dogs (< 1 year-old). As already reported, this could be related to an immature immune system in these dogs making them more vulnerable to infection in their first or second year of life [2, 11, 20, 21]. No significant impact of sex on infection seroprevalence was detected. In a study examining risk factors for canine leishmaniosis in endemic areas, sex did not emerge as a risk factor [22]. In the present cross-sectional study, seroprevalence was found to vary according to animal size and was higher in dogs weighing over 20 kg. The explanation for this could be that a greater weight translates to an increased risk of infection either because of the greater body surface susceptible to sand fly bites or because medium-sized and large dogs are often used for work activities and remain outdoors for long periods of time [20, 22]. No significant relationship was found with factors such as habitat (rural, periurban or urban) or use given to the animal (e.g. hunting, guard, pet) of *L. infantum* infection. It should be noted that the individual immune response of the dog and virulence of the *L. infantum* isolate are important contributing factors [23].

The sand fly vectors of *L. infantum* identified here (*P. perniciosus* and *P. ariasi*) accounted for 88.75% of the total number of specimens identified. The wide distribution of these species, especially of *P. perniciosus* (83.13%), may indicate both an animal health and public health risk. *Phlebotomus ariasi* was distributed in zones of altitudes belonging to the supra-Mediterranean and meso-Mediterranean climates, as this species has a preference for wet and mountainous zones [9, 24–26]. Notwithstanding, it should be underscored that six specimens of this species were found at an altitude of 144 m above the sea level, close to the coast in Carboneras (Almería), although in a study conducted in Hérault (France), 8 individuals were captured at 80 metres of altitude 35 km from the coast [27].

*Phlebotomus papatasi* and *P. sergenti* were detected in the thermo-Mediterranean and meso-Mediterranean belts. This may be due to the fact that both species need environments with some aridity along with warm temperatures for their development [28]. Similarly, these two species are epidemiologically important in other world zones. Thus, *P. papatasi* is a vector of *L. major* in regions of Africa and Asia, while *P. sergenti* is a vector of *L. tropica* in North Africa, causing cutaneous leishmaniosis in both cases [29]. Fortunately, these two species of *Leishmania* have not been detected yet in Spain.

It should be noted that different species of sand flies were found here in places not described to date (*P. ariasi* in Burgos, *P. papatasi* in Huelva and *P. perniciosus* in Segovia). These observations reveal the lack of systematic surveys for the Iberian Peninsula and Balearic Islands, thus generating gaps in the data available or, in other words, a lack of representation of all the species present in a given zone.

According to the statistical treatment of the data, some variables were found to have a significant impact on the density of certain sand fly species. First, a higher density of *S. minuta* was observed in rural habitats. This was expected as this species is clearly a herpetophile [30]. Notwithstanding, in a study by Benito-de Martín et al. [31] in Zaragoza, a greater density of this species was observed in periurban rather than in rural settings.

*Phlebotomus ariasi* was also detected at higher densities in rural habitats. The explanation for this is that this species is exophilic, and consequently, prefers natural environments and daytime resting places far from human settlements [30].

Finally, *P. perniciosus* showed greater densities in the thermo-Mediterranean compared with the meso-Mediterranean belt. While this is a cosmopolitan species that thrives in different bioclimatic zones [2, 30], its preferred thermo-Mediterranean region features a warm climate with long summers allowing for two abundance peaks, one in early summer and the other at the beginning of autumn [2, 32]. In addition, compared with the meso-Mediterranean belt, the thermo-Mediterranean belt also showed significantly higher densities of both vector species (*P. perniciosus* and *P. ariasi*) and total sand fly species. However, this trend noted is clearly biased as *P. perniciosus* represented 83.13% of all species. Phlebotomine vectors of *L. infantum* are sensitive to climate variations, and in the Mediterranean subregion were identified in the period of June-October [32]. Bioclimate predetermines the time needed for completion of sand fly development and life-cycle progression of the parasite within the invertebrate [33].

## Conclusions

This latest knowledge of seroprevalence distributions of canine *L. infantum* infection in Spain defines non-endemic, hypoendemic, endemic and hyperendemic areas. As far as we are aware this is the first time to propose a CanL map for the whole territory of Spain. Along with the distributions of the parasiteʼs sand fly vectors, this information is key to approaching the control of this significant zoonosis. Further, these detailed maps of CanL in Spain including more than three decades (1985–2019) are an important resource for future eco-epidemiological analyses at the national and regional levels. Both bibliography and survey maps combine the information needed for improved management of Canl and could exhibit an accurate prevalence predictive values. The methodology used to build the figure compendium can be extrapolated to other countries or even at the European level.

## Supplementary information

**Additional file 1: Table S1.** Detailed information about sites surveyed for sand flies across the Iberian Peninsula and Balearic Islands.

## Data Availability

All data generated or analysed during this study are included in this published article. Raw data are available from the corresponding author upon reasonable request.

## Abbreviations

CanL: canine leishmaniosis
IFAT: indirect immunofluorescence antibody test
